# Bioinformatics analysis and reveal potential crosstalk genetic and immune relationships between atherosclerosis and periodontitis

**DOI:** 10.1038/s41598-023-37027-x

**Published:** 2023-06-27

**Authors:** Wenyuan Dong, Yuxin Gong, Bin Yang, Bao Li

**Affiliations:** Second hospital of Shanxi medical university, Taiyuan, 030000 China

**Keywords:** Genetics, Immunology, Biomarkers, Cardiology, Diseases

## Abstract

Periodontitis is an inflammatory and immune-related disease with links to several systemic diseases, and the pathological process of atherosclerosis also involves inflammatory and immune involvement. The aim of this study was to investigate the common immune cells and potential crosstalk genes between periodontitis (PD) and atherosclerosis (AS). By analyzing the weighted gene co-expression network of differentially immune infiltrating cells in two diseases to obtain important module genes, and taking the intersection of the module genes, we obtained 14 co-expressed immune-related genes, and evaluated the predictive value of 14 immune-related genes using three machine learning models.Two potential immune-related crosstalk genes (*BTK* and *ITGAL*) were finally obtained by taking intersections of WGCNA intersection genes, DEGs and IRGs.Then, the diagnostic column line graphs were constructed based on the 2 crosstalk genes, and the calibration curves, DCA curves and clinical impact curves indicated that the two genes had strong disease prediction ability, and we further validated the accuracy of the two potential crosstalk genes for disease diagnosis in the validation dataset.Single gene GSEA analysis showed that both genes are jointly involved in biological processes such as antigen presentation and immune regulation, and single sample GSEA analysis showed that macrophages and T cells play an important role in periodontitis in atherosclerosis.This study explored the genetic correlation between atherosclerosis and periodontitis using bioinformatics tools. *BTK* and *ITGAL* were found to be the most important crosstalk genes between the two diseases and may have an important role in the diagnosis and treatment of the diseases. Macrophage and T cell mediated inflammatory and immune responses may play an important role in periodontitis and atherosclerosis.

## Introduction

Periodontitis is an infectious disease caused by multiple factors^1^, Aggregatibacter actinomycetemcomitans and Porphyromonas gingivalis are important pathogens of aggressive periodontitis. Periodontitis can occur at all stages of life, but mainly occurs in adulthood, and 50% of adults suffer from varying degrees of periodontitis^2^. The pathological features of periodontitis include gingival inflammation and irreversible destruction of dental bone and alveolar bone, but the lesions mainly affect specific teeth or tooth surfaces, and the teeth surrounding the lesions are hardly affected^3^. The specificity of this lesion cannot be explained by oral microbial dysbiosis alone, or even by microbial dysbiosis or immunopathology; periodontitis is a systemic disease with a combination of immune and inflammatory effects caused by dysbiosis. Both the innate and adaptive immune systems have an important role in the development and progression of periodontitis^4^. And the presence of periodontitis may affect other inflammatory and immune-related diseases^5^, There is a close relationship between periodontitis and systemic diseases such as cardiovascular disease, type 2 diabetes and rheumatoid arthritis^6^. Three basic mechanisms are currently thought to play a role in the interaction of periodontitis and other diseases, including metastatic infection, inflammation and inflammatory injury, adaptive immunity. Of these, inflammation and inflammatory injury, adaptive immunity play equally important roles in the process of atherosclerosis^7^. Atherosclerosis is an inflammatory and immune-related disease whose incidence is increasing every year and has become a major contributor to death worldwide^8^. Atherosclerosis can lead to a variety of serious complications such as stroke and sudden death^9^. The pathogenesis of atherosclerosis is related to the disorders of lipid metabolism, and a large amount of fat accumulation leads to the formation of plaques. More and more studies have shown that atherosclerosis is closely related to inflammation and immune dysfunction^10^. Macrophages form foam cells after phagocytosis of large amounts of lipids, which accumulate to form the main body of plaque^11^, while a variety of immune cells play a role in the formation of plaque. Periodontitis has been shown to be a promoter of plaque formation in experiments with animal models of atherosclerosis^12^. Periodontitis causes immune cells in atherosclerotic plaques to activate and release large amounts of inflammatory factors, such as interferon, interleukin-1, and TNF-$\alpha$, which induce IL-6 production, IL-6 stimulates the production of acute phase reactants such as CRP, which leads to the development of local inflammation and promotes the formation of atherosclerotic plaques^13^. Studies have found that major depression, physical inactivity, cardiovascular disease and family history of periodontal disease, age and gender are all secondary risk factors for atherosclerotic cardiovascular disease, and these factors are also common in patients with periodontitis^14^. At present, the relationship between periodontitis and atherosclerosis still needs further study. Inflammatory response and immune regulation are the key pathogenic factors of both diseases. With the public availability of sequencing data of periodontitis and atherosclerosis, we investigated the relationship between the two diseases and immune cells by bioinformatics, and found the immune crosstalk genes between the two diseases, assessed the efficacy of these two genes in predicting the disease and diagnosing the disease, and provided a reference for further investigate the pathophysiological process of periodontitis and atherosclerosis.

## Materials and methods

### Data download

We obtained gene expression data for periodontitis and atherosclerosis from the GEO database. The dataset GSE16134 about periodontitis was used as the experimental set and GSE10334 as the validation set; the dataset GSE100927 about atherosclerosis was used as the experimental set and GSE43292 as the validation set. Immune-related genes (IRGs) were obtained from the Immport database (https://www.immport.org/shared/home).

### Immune cell infiltration analysis

The relative abundance of 22 immune cells was calculated using the CIBERSORT algorithm and the LM22 gene set of immune cells in periodontitis samples and atherosclerosis samples^15^, respectively, and after excluding samples with statistically insignificant results (p value> 0.05), the Wilcoxon test was used to compare the differences in 22 immune cells between samples from the normal and disease groups.

### Weighted gene co-expression network analysis

The WGCNA contains genes with adjusted P-values < 0.05. Hierarchical clustering was first performed using the “Hculst” function in R to assess whether there were significant outliers^16^. Next, the “pickSoftThreshold” function was used to select the appropriate soft threshold power /beta/ to make the gene expression relationships conform to the scale-free network. WGCNA was used to analyse the association patterns of different samples with 22 immune cells and to cluster genes with similar expression patterns and analyse the association of specific modules with immune cells^17^. Co-expression networks were constructed using the WGCNA R package and the modules with the highest correlation with differential immune cells (|correlation coefficient|>0.5, p-value <0.05) were extracted. Genes with |MM|>0.8 and |GS|>0.4 in the modules were considered as key module genes. MM indicates the correlation of genes with modules and GS indicates the correlation of genes with traits. Each gene has a corresponding MM and GS value, with a higher MM value indicating that the gene is more relevant to a module in the WGCNA, and a higher GS value indicating that the gene is more important in a module in the WGCNA. Two modules of interest were selected from each of periodontitis and atherosclerosis, and immune-related co-expressed genes were obtained after taking the intersection.

### Identification of DEGs

The original expression matrix was normalized using R software, and differentially expressed heterozygotes were screened using the limma package with the following screening criteria: the threshold was |log2FoldChange (FC)| > 1 and adjusted p value < 0.05. After obtaining the differential genes, the top 50 differential genes were mapped into a differential gene volcano map using R software.

### Machine learning model screens for crosstalk genes

We obtained 14 co-expressed immune cell-associated genes by taking the intersection using WGCNA of two diseases. Based on these 14 immune-related genes, three machine learning models were constructed using the caret’R package: a random forest model (RF), a support vector machine model (SVM), and a generalized linear model (GLM). Using the 14 immune cell-associated genes as explanatory variables and the presence or absence of disease as a response variable. The three models were then analyzed using the “interpretation” function in the “DALEX” R package and the cumulative residual distribution was plotted to obtain the best model. Finally, we analyzed the importance of these immune-related genes in predicting response variables (normal or diseased).

### Construction and validation of a nomogram

We obtained two immune-related differentially expressed crosstalk genes by taking the intersection of WGCNA co-expression genes, DEGs and IRGs, and applied the “RMS” package to create a column line graph based on the two crosstalk genes for clinical assessment of atherosclerosis and periodontitis. The predictive accuracy of the line graph was assessed by plotting calibration curves. The clinical value of the line graph was evaluated by plotting decision curves and clinical impact curves.

### ssGSEA

The “GSVA” R package was used for ssGSEA, and the infiltration of 22 immune cells in pathological and normal samples was analyzed. In order to study the correlation between core genes and the abundance of infiltrating immune cells, The criterion for screening was p-value < 0.05.

### Candidate biomarker expression levels and diagnostic value

We further validated the expression levels of hub genes in the validation set using the R software ggplot2 package to plot box plots (p < 0.05). The validity of potential biomarkers was also assessed by the area under the curve (AUC) using the pROC R package to plot the subject operating characteristic (ROC) curves of the two hub genes in the four data sets.

## Results

### Immune cell infiltration in two diseases

**Figure 1:**
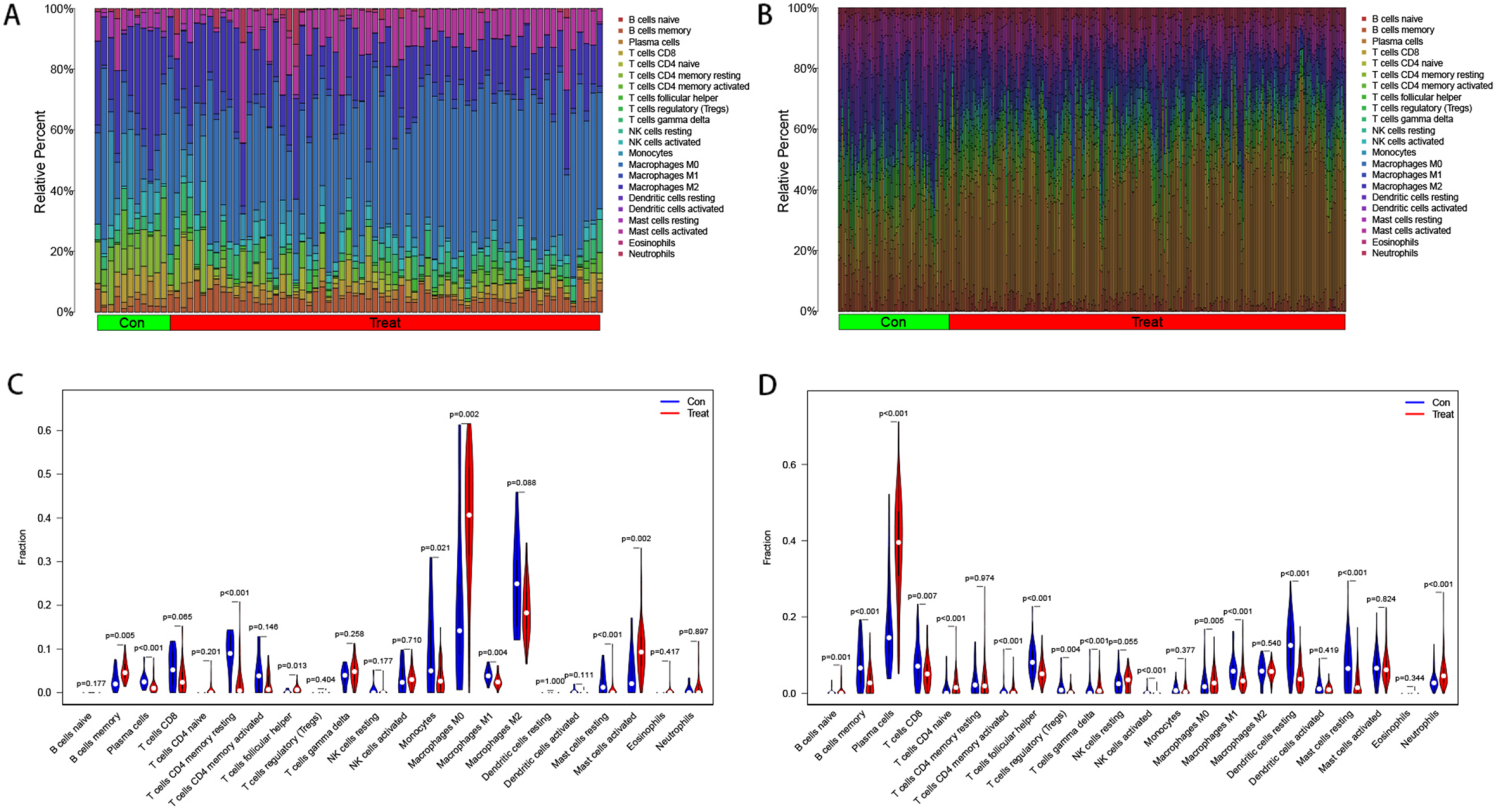
Analysis of immune infiltration in atherosclerosis and periodontitis.Barplot (**A**) and vioplot (**C**) show the distribution of 22 immune cells in atherosclerotic samples. Barplot (**B**) and vioplot (**D**) show the distribution of 22 immune cells in periodontitis samples. *Con* control; *Treat* diseases; P < 0.05;**P < 0.01; ***P < 0.001.

We calculated the infiltration of 22 immune cells in each of the two diseases using the CIBERSORT algorithm, and Fig. 1A, B shows the percentage of immune cell infiltration in atherosclerosis and periodontitis, respectively. As shown in Fig. 1C, D, the proportion of Macrophages M0, B cells memory and Mast cells activated was significantly higher in atherosclerotic samples compared to normal samples. In periodontitis samples, Plasma cells, Neutrophils, T cells CD4 naive, Macrophages M0 were significantly increased compared to normal samples. Among them, T cells CD4 naive and Macrophages M0 infiltration was significantly increased in both diseases.

### WGCNA filters for immune cell-related genes

**Figure 2:**
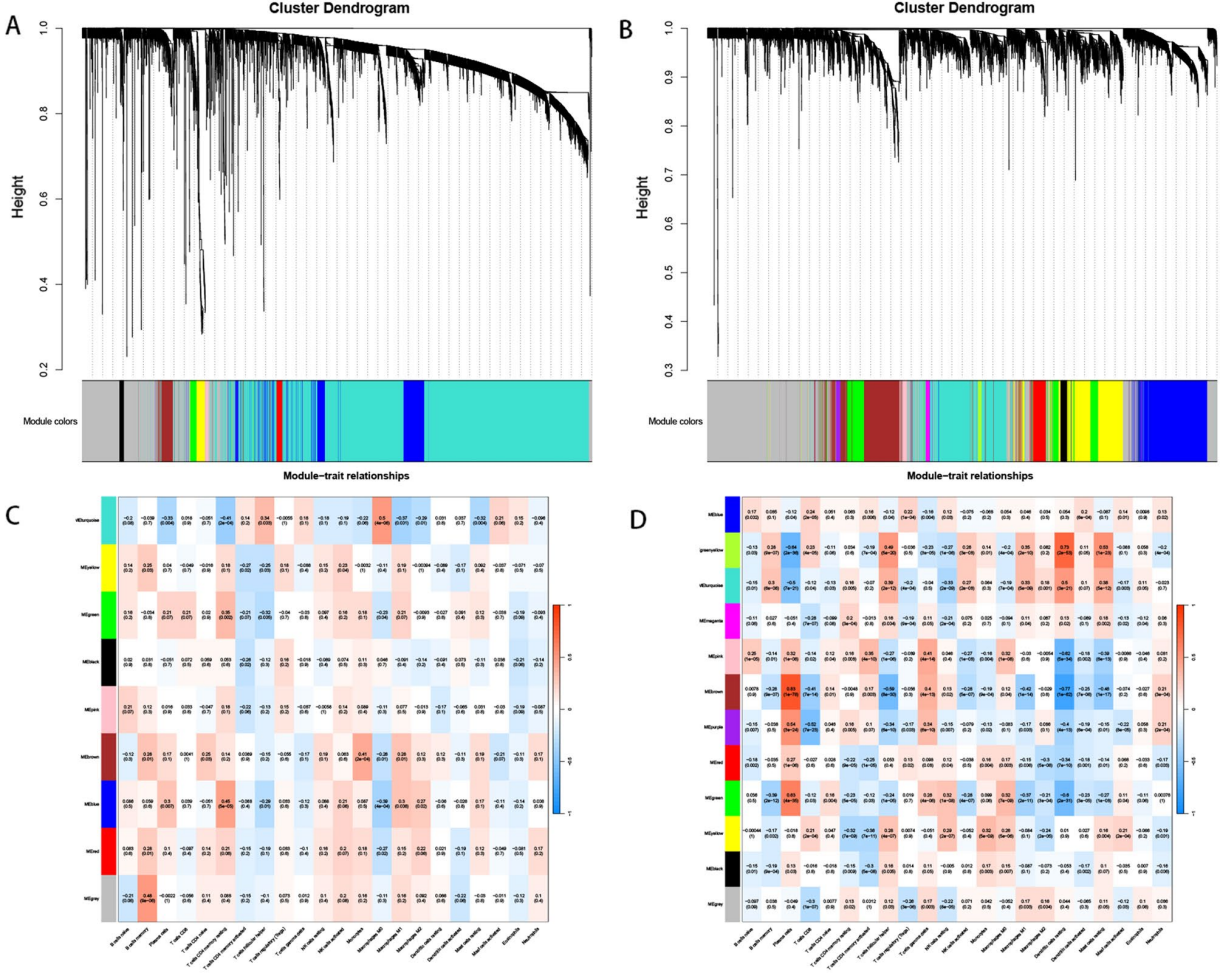
Weighted gene co-expression network analysis based on differential immune infiltration cells. Cluster dendrogram (**A**) and heatmap (**C**) show the correlation between modules and immune cells in atherosclerotic disease. Cluster dendrogram (**B**) and heatmap (**D**) show the correlation between modules and immune cells in periodontitis disease.

We performed WGCNA analysis using the results of the infiltration of expressed genes in immune cells for each of the two diseases, without deleting samples in the sample clustering, and the soft-thresholding power $\beta = 13$ (scale-free R2 $=$ 0.9) was chosen in the WGCNA of atherosclerotic samples to construct a scale-free network (Supplementary Fig. 1A). The soft-thresholding power $\beta = 15$ (scale-free R2 $=$ 0.9) was selected in WGCNA of periodontitis samples to construct a scale-free network (Supplementary Fig. 1B). Afterwards, the cluster dendrogram was constructed separately and the modules were merged (Fig. 2A,B), and the correlation between different modules and immune cells was assessed by plotting heatmaps (Fig. 2C,D). A total of 9 modules were obtained from the atherosclerosis sample, and the highest positive correlations were selected from the turquoise and Macrophages M0 modules, and the highest negative correlations were selected from the turquoise and T cells CD4 memory resting modules. A total of 12 modules were obtained from the periodontitis samples, and the brown and Plasma cells modules with the highest positive correlation, and the brown and Dendritic cells resting modules with the highest negative correlation, were selected from among them. Among these key modules, the key genes were screened with |MM|> 0.8 and |GS| > 0.4^18^, and then the key genes in the four modules were taken as intersection (Supplementary Fig. 2A–2D) to obtain a total of 14 immune-related co-expressed genes (Fig. 4E).

### Machine learning models evaluate biomarkers

**Figure 3:**
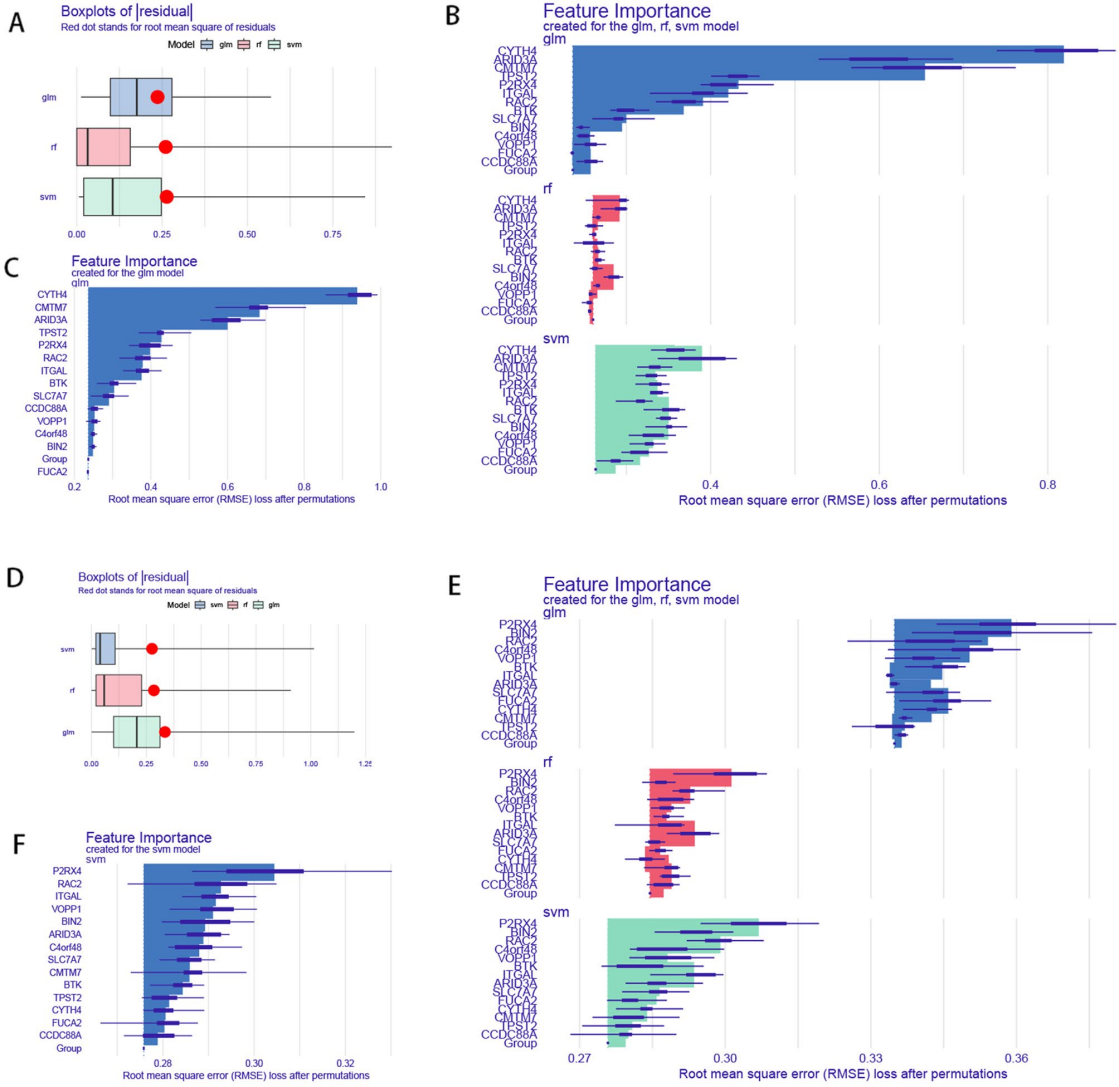
Using machine learning to build models of disease diagnosis. In the atherosclerotic sample, (**A**) boxplot of sample residuals. (**B**) Significance of variables in the RF, GLM, and SVM models. (**C**) Significance of variables in the GLM model. In the periodontitis sample, (**D**) boxplot of sample residuals. (**E**) Significance of variables in RF, GLM, and SVM models. (**F**) Significance of variables in the SVM model.

We used three machine learning models: random forest model (RF), support vector machine model (SVM), and generalized linear model (GLM) to evaluate the importance of each of the 14 immune-related genes for disease prediction. As shown in Fig. 3, the residual distributions (Supplementary Fig. 4A) and boxplot (Fig. 3A) of the three models in atherosclerotic disease were plotted using the explanatory feature function of the DALEX package, and the results showed that the GLM model had the best predictive effect. We then used 14 genes as variables to predict their importance in the response variable (disease or not), and found that all 13 genes except *CCDC88A* had good predictive value (Fig. 3B,C). And in periodontitis, we constructed a disease prediction model using the same method to construct residual distribution plots (Supplementary Fig. 4B) and box line plots, and the results showed that the SVM model had the best prediction (Fig. 3D) and all 14 genes had good disease prediction value (Fig. 3E,F).

### Identification of crosstalk genes

**Figure 4:**
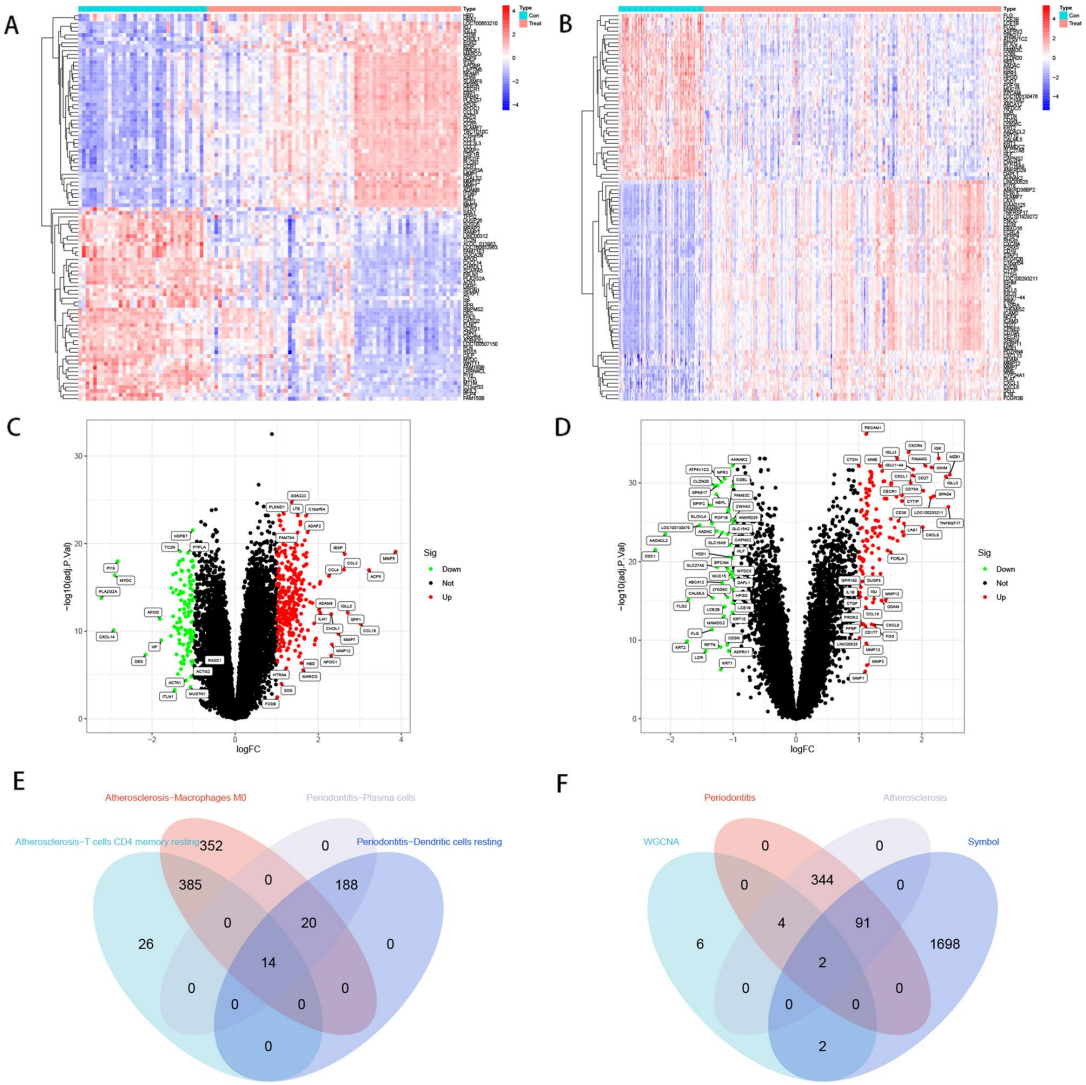
Acquisition of differentially expressed genes. (**A**) Heatmap showing the top 50 DEGs expressed in atherosclerotic samples. (**B**) Heatmap showing the top 50 DEGs expressed in periodontitis samples. (**C**) Volcano plot showing DEGs in atherosclerotic samples. (**D**) Volcano plot showing DEGs in periodontitis samples. (**E**) Venn shows intersecting genes of the WGCNA module in atherosclerosis and periodontitis. (**F**) Venn shows 2 core genes common to WGCNA and DEGs and IRGs. *Con* control; *Treat* diseases; *DEG* differentially expressed gene; *WGCNA* weighted gene co-expression network analysis; *IRGs* immune-related genes.

In the periodontitis and atherosclerosis datasets, we screened differentially expressed genes (DEGs) using the limma package, and a total of 421 differentially expressed genes were screened in the atherosclerosis dataset and 167 differentially expressed genes were screened in the periodontitis dataset, and heatmaps of the top 50 differentially expressed genes were drawn using R software (Fig. 4A,B), as well as volcano plots of differential expression in the two diseases (Fig. 4C,D). Two immune-related co-expressed differential genes (*BTK* and *ITGAL*) were then obtained by taking intersections of WGCNA immune-related genes, DEGs and IRGs (Fig. 4F). We further analyzed the correlation between the two genes and 22 immune cells (Supplementary Fig. 3A–3D), and the results showed that in atherosclerosis and periodontitis, Macrophage M0 and CD4+ T cells are positively correlated with crosstalk genes. We also analyzed the biological processes involved in crosstalk genes by ssGSEA. GO enrichment analysis showed that the two genes were mainly involved in antigen presentation and immune regulation in BP, As to CC, two genes are mainly related to the endoplasmic reticulum. As to MF, two genes are mainly involved in antigen binding, and KEGG enrichment analysis showed that two genes were mainly enriched in B cell receptor signaling pathway and Primary immunodeficiency (Supplementary Table 1)^19^.

### Construction a nomogram for diagnosis

**Figure 5:**
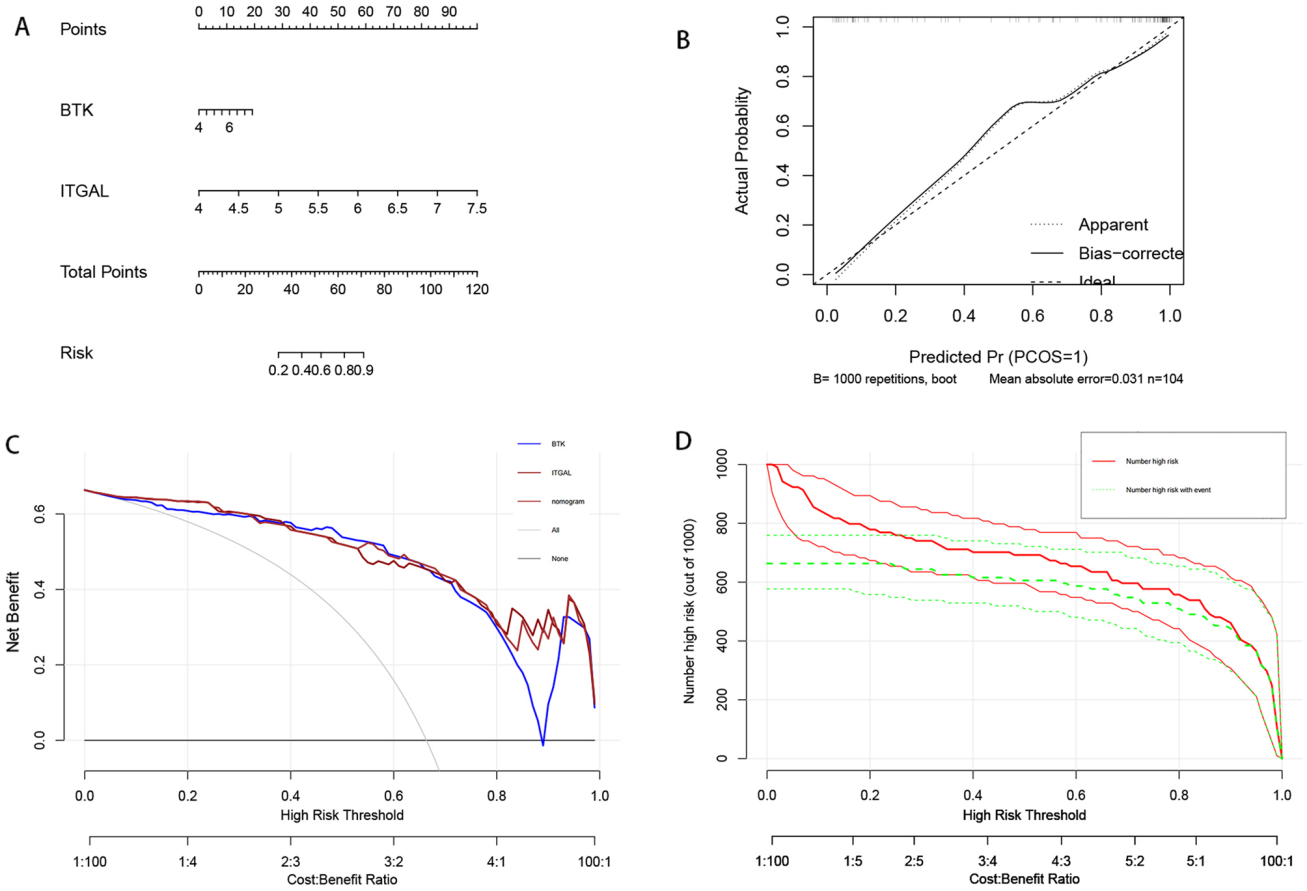
Construction and validation of a nomogram model for atherosclerosis diagnosis. (**A**) Nomogram of diagnostic biomarkers for the diagnosis of atherogenesis. (**B**) Evaluation of the predictive ability of the column line graph model by calibration curves. (**C**) Evaluation of the clinical application value of the columnar line graph model using DCA curves. (**D**) Clinical impact curves of the nomogram.

**Figure 6:**
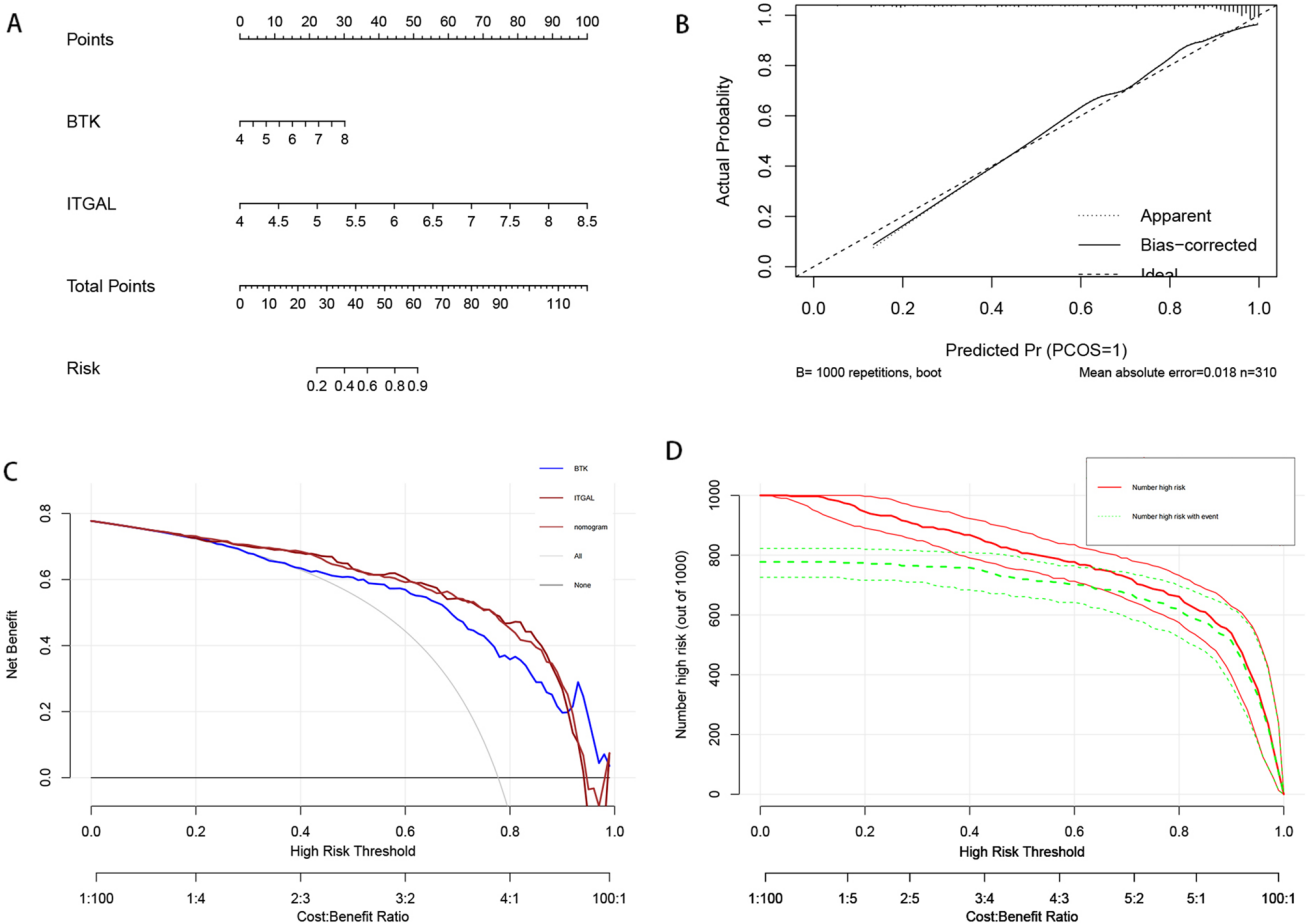
Construction and validation of a nomogram model for periodontitis diagnosis. (**A**) Nomogram of diagnostic biomarkers for the diagnosis of atherogenesis. (**B**) Evaluation of the predictive ability of the column line graph model by calibration curves. (**C**) Evaluation of the clinical application value of the columnar line graph model using DCA curves. (**D**) Clinical impact curves of the nomogram.

After two crosstalk genes were obtained by screening, a nomogram based on the two crosstalk genes was built using the “RMS” package in each of the two diseases (Figs. 5, 6). In atherosclerosis disease, we constructed a nomogram prediction model (Fig. 5A) and evaluated the prediction accuracy of the nomogram using calibration curves (Fig. 5B), which showed that the error between the actual risk of atherosclerosis and the predicted risk was small, indicating that the prediction of the column line graph was accurate. As shown in Figure 5C, the nomogram curve is higher between 0.5 and 1 than the other curves, indicating that patients can benefit from a columnar plot with a high risk threshold. Clinical impact curves showed that Number high risk curve and Number high risk with event curve tended to be close at 0.4–1, indicating that nomogram has better predictive ability (Fig. 5D). In periodontitis, we similarly constructed the nomogram for predicting the occurrence of periodontitis (Fig. 6A), and The calibration curve showed the high predictive accuracy of the nomogram (Fig. 6B). The DCA curve shows that patients can benefit from nomogram to predict diagnosis in the range of 0.8–1 (Fig. 6C), and clinical impact curves show that Number high risk curve and Number high risk with event curve tend to be close at 0.4–1, indicating that nomogram has good predictive ability (Fig. 6D). Both of these results suggest that both crosstalk genes play an important role in the development of periodontitis and atherosclerosis.

### Expression levels and diagnostic value of candidate biomarkers

**Figure 7:**
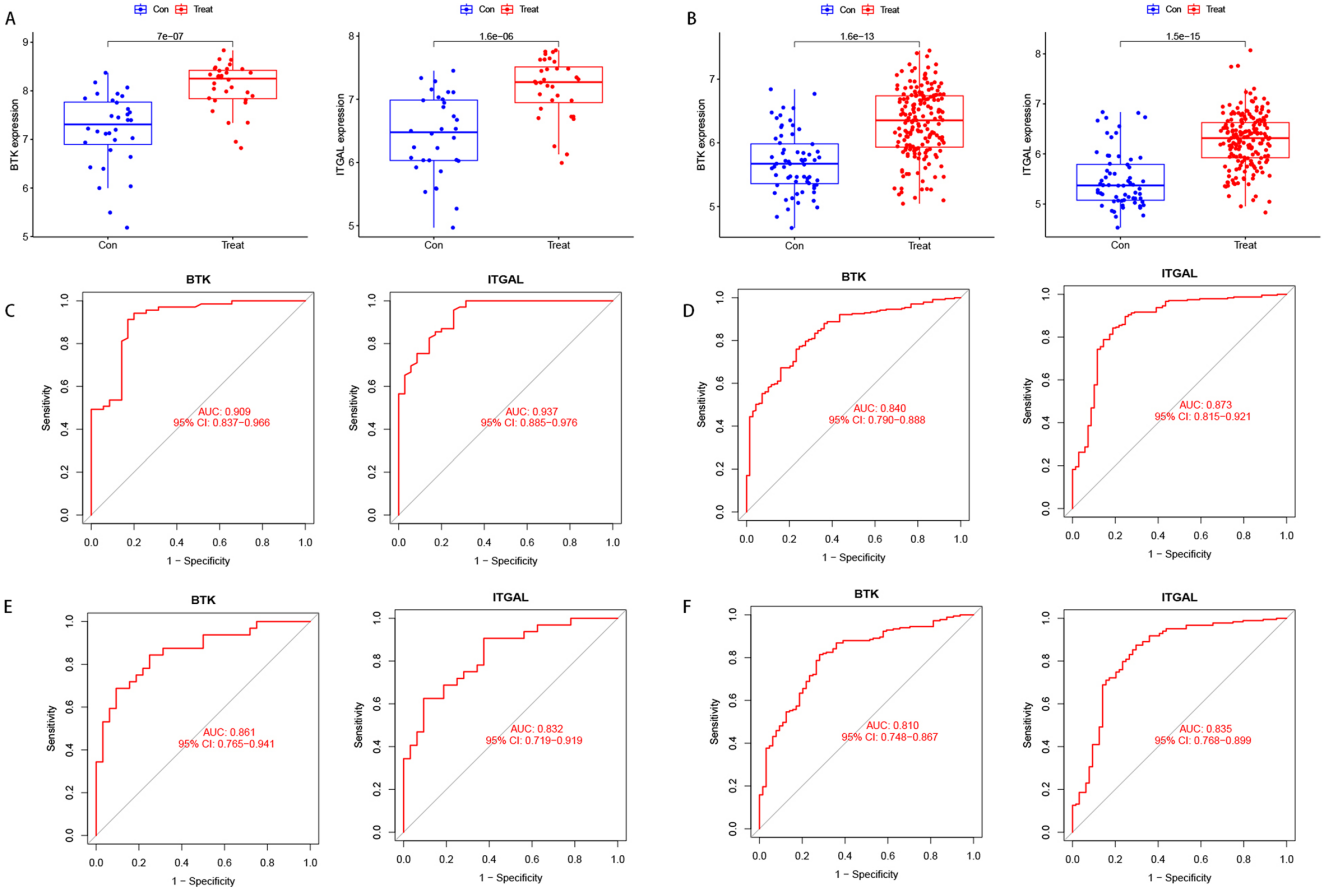
Expression pattern validation and diagnostic value. (**A**) Expression of *BTK* and *ITGAL* in GSE43292. (**B**) Expression of *BTK* and *ITGAL* in GSE10334. (**C**) ROC curve of the shared diagnostic genes in GSE100927. (**D**) ROC curve of the shared diagnostic genes in GSE16134. (**E**) ROC curve of the shared diagnostic genes in GSE43292. (**F**) ROC curve of the shared diagnostic genes in GSE10334. *Con* control, *Treat* diseases.

We verified the expression levels of two crosstalk genes separately in the validation dataset (Fig. 7A,B), and the results showed that *BTK* and *ITGAL* were upregulated in both atherosclerosis and periodontitis. We further evaluated the diagnostic sensitivity and specificity of both genes by ROC curves, and both genes had good diagnostic value in the experimental set (Fig. 7C,D): *BTK* (AUC = 0.909) and *ITGAL* (AUC = 0.937) in atherosclerosis; *BTK*(AUC = 0.840) and *ITGAL*(AUC = 0.873)in periodontitis. We then further validated the diagnostic effect of both genes in the validation dataset (Fig. 7E,F): *BTK* (AUC = 0.861) and *ITGAL* (AUC = 0.832) in atherosclerosis; and *BTK* (AUC = 0.810) and *ITGAL* (AUC = 0.835) in periodontitis. Both results indicated good predictive power of both genes.

## Discussion

Both periodontitis and atherosclerosis belong to NCDs, the incidence of which has been on the rise in recent years, among which cardiovascular disease has the highest morbidity in the entire world. Atherosclerosis is an important factor causing cardiovascular diseases. Periodontitis is the sixth most common human disease worldwide, There is substantial evidence that periodontitis is associated with cardiovascular disease^20^, diabetes^21^, chronic obstructive pulmonary disease^22^, chronic kidney disease^23^. Among them, severe periodontitis was independently and significantly associated with all-cause mortality and cardiovascular mortality^24^. Evidence from epidemiological studies suggests that patients with periodontitis exhibit significant endothelial dysfunction, which leads to a significant increase in the incidence of atherosclerosis. Antibodies to periodontal pathogens may play an important role in periodontitis and atherosclerosis, de Boer et al. find that antibodies to periodontal pathogens are associated with coronary plaque remodeling^25^. The same results were obtained in experiments with animal models^26^. Through ssGSEA analysis, we also found that crosser genes are mainly concentrated in antigen presentation, antigen binding and immune regulation.

In this study, We used the datasets of atherosclerosis and periodontitis for our analysis,all included datasets were batch corrected using the SVA package, and outliers were removed.The results suggested that cross-platform normalization successfully eliminated the batch effect.Then we analyzed the expression datasets of periodontitis and atherosclerosis for the first time using WGCNA combined with immune cell infiltration, identified potential immune-related crosstalk genes and common immune cells in both diseases. We identified *SLC7A7*, *BTK*, *CYTH4*, *BIN2*, *ITGAL*, *P2RX4*, *C4orf48*, *CMTM7*, *TPST2*, *RAC2*, *ARID3A*, *VOPP1*, *FUCA2* and *CCDC88A* as important crosstalk genes in periodontitis and atherosclerosis, three machine learning methods were used to build diagnostic models and evaluate the value of the 14 crosstalk genes for disease diagnosis. FUCA2 is not effective in the diagnosis of atherosclerosis. *SLC7A7*, *BTK*, *CYTH4*, *BIN2*, *ITGAL*, *P2RX4*, *C4orf48*, *CMTM7*, *TPST2*, *RAC2*, *ARID3A*, *VOPP1*, *FUCA2* and *CCDC88A* have good diagnostic value in both diseases. We then obtained two immune-related differentially expressed genes through the intersection of 14 WGCNA genes, DGEs and IRGs. By establishing a clinical diagnostic model, the accuracy of the two crossers for disease diagnosis was further evaluated. In addition, the diagnostic accuracy of the two crossers was verified in the validation dataset. The results showed that *BTK* and *ITGAL* had good diagnostic value as diagnostic genes for two diseases. First of all, we performed an immune cell infiltration analysis on the expression profile data of both diseases and found that T cells and Macrophages infiltrated significantly more in both diseases. Macrophages are derived from monocytes and play an important role in defending against bacterial infections and regulating the immune defense process^27^. M0 macrophages are induced to differentiate under different conditions to form M1 and M2 macrophages, of which M1 cells have pro-inflammatory functions while M2 cells have anti-inflammatory functions. Macrophages mainly play a regulatory and phagocytic role in periodontitis, and have an important role in maintaining periodontal tissue homeostasis and defense^28^. However, over-activated macrophages produce large amounts of pro-inflammatory factors that cause inflammatory and immune responses that can damage periodontal tissues. Macrophages will show different responses to stimuli from different pathogens and microorganisms, leading to a diversity of chronic inflammatory conditions^29^. Interconversion between M1 and M2 phenotypes of macrophages may be an important mechanism leading to periodontal tissue damage^30^. Macrophage dysfunction is the basis of atherogenesis and plays an important role in all stages of the lesion^31^. Atherosclerosis is an inflammatory disease caused by endothelial damage of blood vessels, where large amounts of cholesterol, lipids and cellular debris are deposited in the vessel walls leading to plaque formation. And macrophages and foam cells play an important role in the formation of plaque^32^. Macrophages will absorb the excessive accumulation of cholesterol and lipids at the lesion site. When the absorption of macrophages exceeds their excretion, the free cholesterol will be converted into cholesterol lipids and accumulated in the cells, resulting in the transformation of macrophages into foam cells. Activation of macrophages activates T cells through antigen presentation, and CD4+ T cells are the main lymphocytes in the fight against periodontitis^33^, Macrophages can be involved in host immunity by way of secreting cytokines. An important link between T cells and alveolar bone loss has also been shown^34^. The relationship between T cells and atherosclerosis has also been paid more and more attention. It has been found that T cells are mainly distributed in the fibrous cap of plaques in human and mouse lesions^35^, Interaction between antigen-presenting cells (APCs) and CD4+T cells in atherosclerotic plaques has been found to result in secretion of many pro-inflammatory cytokines, and depletion of CD4+T cells through genetic pathways or antibody binding can inhibit the progression of lesions in mouse models of atherosclerosis^36^. Then, through the intersection of WGCNA, DEGs and IRGs, we screened the co-expressed immune-related crossers *BTK* and *ITGAL* of the two diseases. Bruton Tyrosine Kinase (*BTK*) belongs to the nonreceptor Tec tyrosine kinase family and is mainly expressed in lymphocytes and myeloid cells^37^. *BTK* can mediate inflammation and cell differentiation, in addition, *BTK* is expressed in osteoblasts and may have a role in regulating osteoblast proliferation and differentiation. Masahiro et al. found that the *BTK* inhibitor PCI-32765 inhibited osteoclast differentiation in mice with osteoporosis. In periodontitis, *BTK* can aggravate periodontal bone destruction by promoting osteoclast differentiation^38^. *BTK* inhibitors are expected to be potential drugs for treating periodontitis. The NF-/kappa/ B signaling pathway plays an important role in atherosclerosis^39^, It can accelerate plaque formation through pro-inflammatory effects, and *BTK* can activate the NF-/kappa/ B pathway to promote inflammation as well as arterial plaque formation^40^. In addition, *BTK* is closely related to macrophage-induced oxidative stress, endoplasmic reticulum stress and inflammation. Down-regulation of *BTK* can inhibit the activation of NK-/kappa/ B signaling pathway in ox-LDL-induced macrophages and inhibit M1 polarization. ox-LDL-induced ER stress, oxidative stress, and inflammatory responses in macrophages were also inhibited. *BTK* inhibitors acalabrutinib and ONO/GS-4059 inhibit platelet aggregation during atherosclerosis^41^. *BTK* inhibitors may be potential agents for the treatment of atherosclerosis by regulating macrophage polarization, phagocytosis and secretion of pro-inflammatory factors. The *ITGAL* gene encodes the L chain of integrin /alpha/, a dimer membrane protein composed of /alpha/ and /beta/ chains. Integrin was expressed on all leukocytes^42^. It plays a role in leukocyte adhesion through interaction with ICAMs 1-3(intercellular adhesion molecules 1–3), as well as lymphocyte costimulatory signal transduction. *ITGAL* has been less studied in periodontitis and atherosclerosis. We found that *ITGAL* showed a significant positive correlation with Macrophages M0 and T cells gamma delta in both periodontitis and atherosclerosis samples, suggesting that *ITGAL* plays an equal role in the immune regulation of both diseases.

## Conclusion

This study is the first to explore the potential relationship between periodontitis and atherosclerotic disease using WGCNA. *BTK* and *ITGAL* were found to be the most important cross-signaling genes between periodontitis and atherosclerosis by WGCNA combined with immune cell analysis. T cells and macrophage-driven immune responses may play an important role in periodontitis and atherosclerosis. At the same time, a clinical diagnostic model was established to evaluate the effectiveness of the two genes in disease diagnosis. The inadequacy is that we lack of consideration of confounding variables: the samples included did not take into account potential confounding variables such as age, gender or medication, which may affect gene expression and immune cell infiltration.We will consider and control for these confounding factors in future studies.

## Supplementary Information

Supplementary Figure 1.

Supplementary Table 1.

Supplementary Figure 2.

Supplementary Figure 2.

Supplementary Figure 2.

Supplementary Figure 2.

Supplementary Figure 3.

Supplementary Figure 3.

Supplementary Figure 3.

Supplementary Figure 3.

Supplementary Figure 4.

Supplementary Figure 4.

## Data Availability

The original contributions presented in this study are included in the article/supplementary material. Further inquiries can be directed to the corresponding author.
