# Supplementary material for: Bioinformatics analysis and reveal potential crosstalk genetic and immune relationships between atherosclerosis and periodontitis
Source: Sci Rep. 2023 Jun 27;13:10381. doi: 10.1038/s41598-023-37027-x (PMC10300131; doi:10.1038/s41598-023-37027-x)
Supplement: Supplementary file 1 — Supplementary Figure 1. [file 41598_2023_37027_MOESM1_ESM.pdf]

# Soft threshold and Mean Connectivity

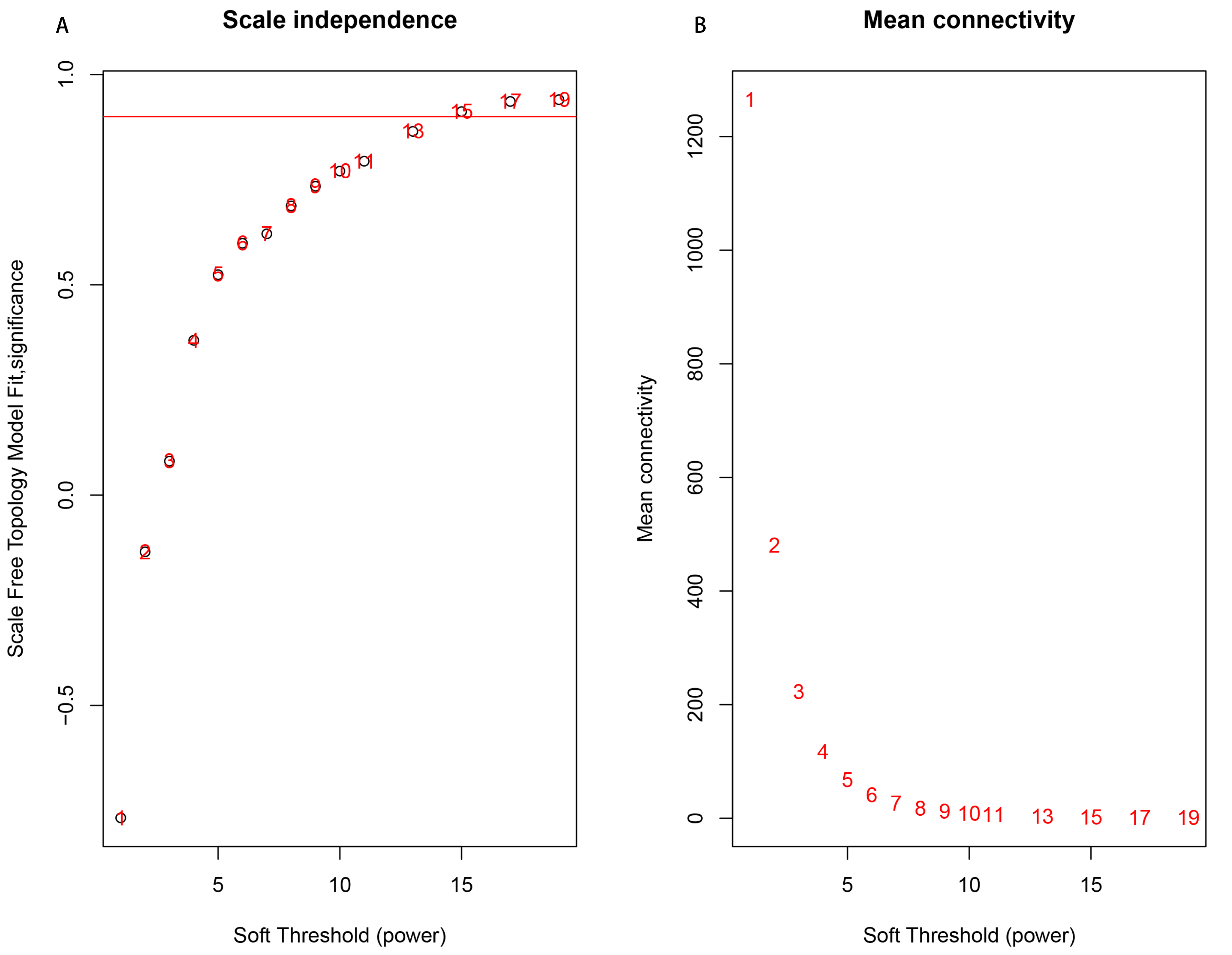


Soft threshold selection

A， A value above 0.9 is chosen as the soft threshold, and the first value reaching above 0.9 is 15. B， At a value of 15, the curve starts to flatten out, indicating that the connectivity of the network is good at a soft threshold of 15.
