# Supplementary material for: Bioinformatics analysis and reveal potential crosstalk genetic and immune relationships between atherosclerosis and periodontitis
Source: Sci Rep. 2023 Jun 27;13:10381. doi: 10.1038/s41598-023-37027-x (PMC10300131; doi:10.1038/s41598-023-37027-x)
Supplement: Supplementary file 3 — Supplementary Figure 2. [file 41598_2023_37027_MOESM3_ESM.pdf]

Screening for differential genes

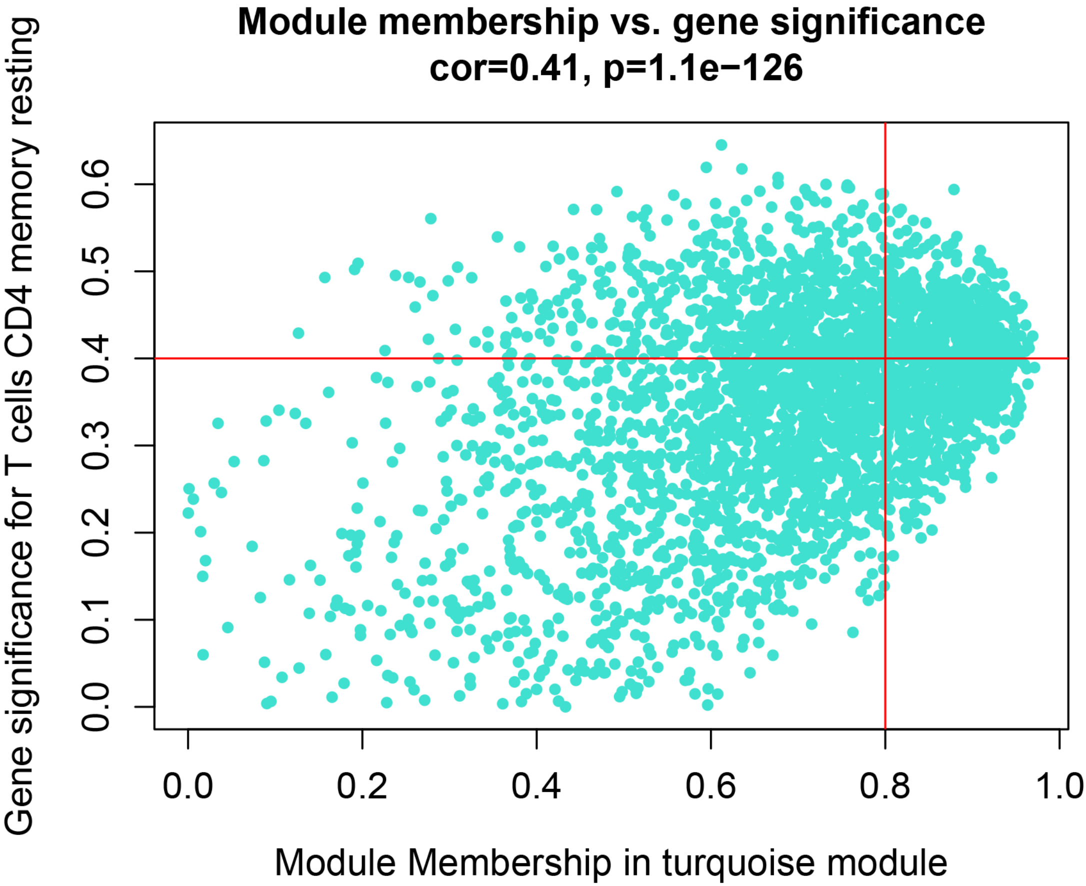

In atherosclerosis interested in WGCNA analysis results of module differences in genetic screening, screening conditions for |MM|>0.8 and |GS|>0.4.
