# Supplementary material for: Bioinformatics analysis and reveal potential crosstalk genetic and immune relationships between atherosclerosis and periodontitis
Source: Sci Rep. 2023 Jun 27;13:10381. doi: 10.1038/s41598-023-37027-x (PMC10300131; doi:10.1038/s41598-023-37027-x)
Supplement: Supplementary file 6 — Supplementary Figure 2. [file 41598_2023_37027_MOESM6_ESM.pdf]

Screening for differential

Gene significance for Dendritic cells resting

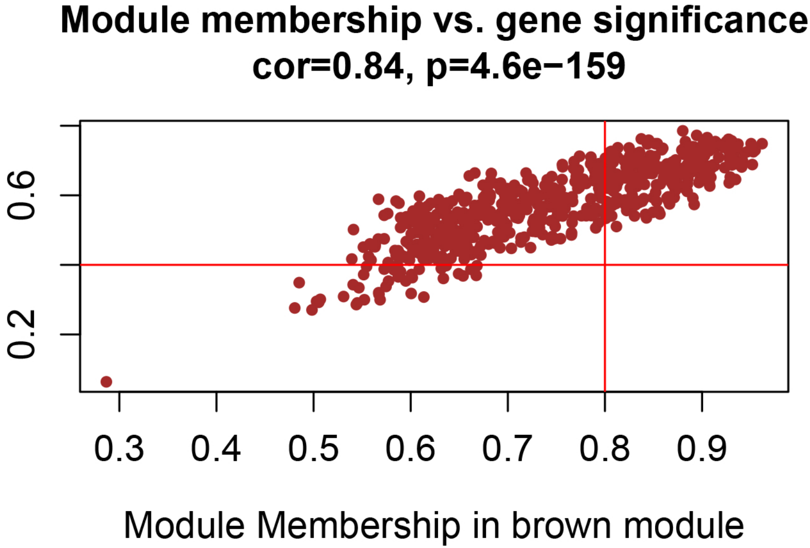

Differential gene screening for modules of interest obtained by WGCNA analysis in periodontitis, and the screening conditions are |MM|>0.8 and |GS|>0.4.
