# Supplementary material for: Bioinformatics analysis and reveal potential crosstalk genetic and immune relationships between atherosclerosis and periodontitis
Source: Sci Rep. 2023 Jun 27;13:10381. doi: 10.1038/s41598-023-37027-x (PMC10300131; doi:10.1038/s41598-023-37027-x)
Supplement: Supplementary file 8 — Supplementary Figure 3. [file 41598_2023_37027_MOESM8_ESM.pdf]

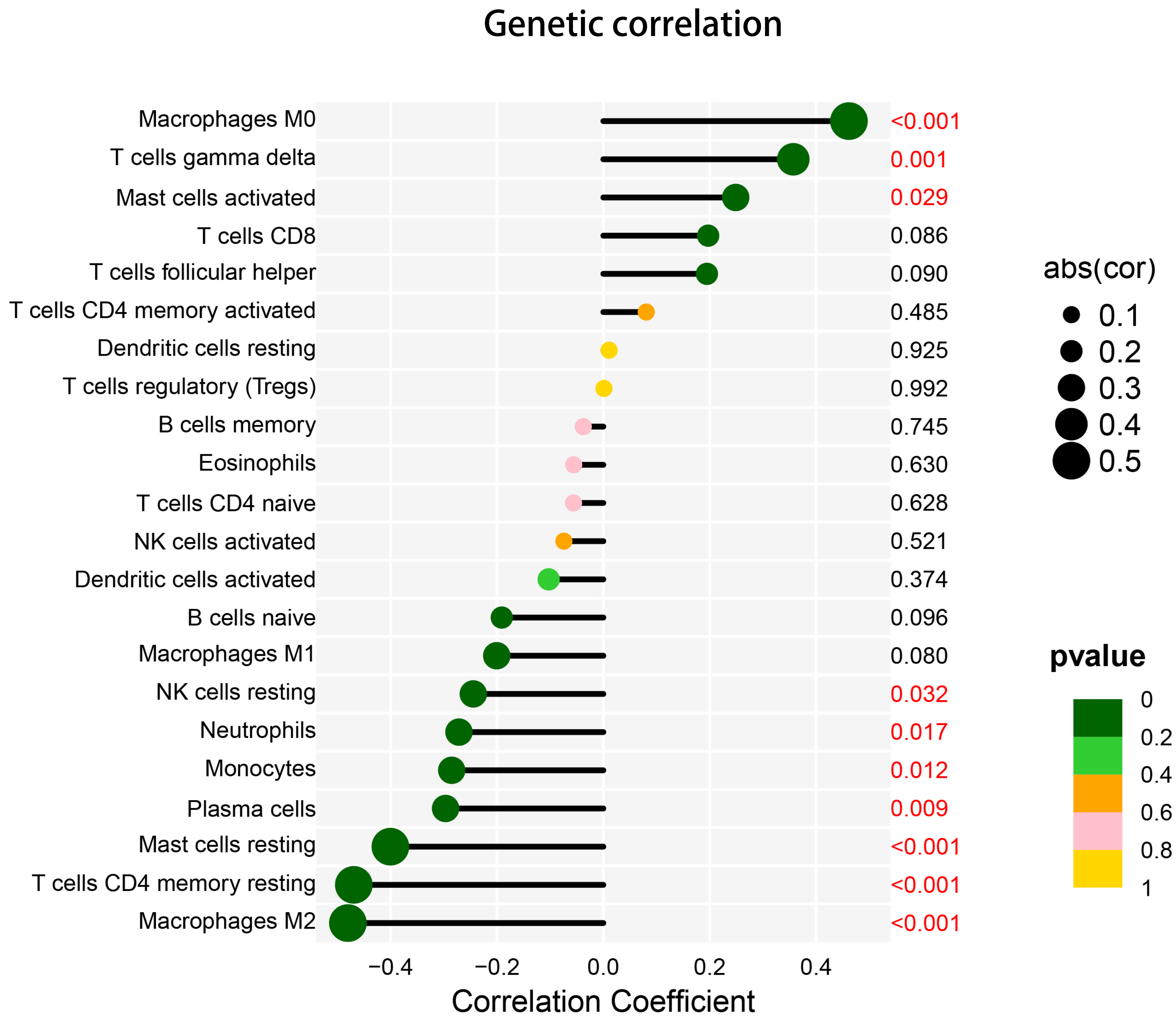

Correlation analysis between ITGAL and 22 kinds of immune cells in atherosclerosis
